# Cropping System Diversification Influences Soil Microbial Diversity in Subtropical Dryland Farming Systems

**DOI:** 10.1007/s00248-022-02074-w

**Published:** 2022-07-15

**Authors:** Alwyn Williams, Henry W. G. Birt, Anil Raghavendra, Paul G. Dennis

**Affiliations:** 1 School of Agriculture and Food Sciences, The University of Queensland, Gatton, QLD 4343 Australia; 2 School of Earth and Environmental Sciences, The University of Queensland, Brisbane, QLD 4072 Australia; 3 New South Wales Department of Primary Industries, Elizabeth Macarthur Agricultural Institute, Menangle, NSW 2568 Australia

**Keywords:** Bacteria, Crop rotation, Fungi, Microbiome, Phylogenetic marker gene sequencing, Soil health

## Abstract

Conventional dryland cropping systems are characterised by low crop diversity and frequent fallows. This has significant impacts on soil microbes that underpin soil function. Diversifying crop rotations can potentially counter these effects; however, limited data exists on the impacts of diversified crop rotations on soil microbes in drylands. Using phylogenetic marker gene sequencing, we characterised soil microbial diversity in conventional and diversified dryland crop rotations in subtropical Australia. This included winter and summer dominant rotations. Conventional systems were cereal-dominant with a crop-fallow rotation. Diversified systems included greater crop diversity, double crops, cover crops, and a multi-year ley pasture. In summer rotations with increased crop diversity and cover crops, bacterial and fungal richness increased, and distinct communities were formed compared to fallow land. Often, these community shifts were associated with greater soil organic carbon (SOC) and nitrogen. All winter rotations had distinct fungal communities and ley pasture resulted in greater fungal diversity compared to other rotations. No effects of the winter rotations were evident on bacterial communities. Our results show that diversification of dryland crop rotations leads to significant shifts in soil microbial communities in both winter and summer cropping systems. Both summer and winter rotations incorporating cover crops and ley pasture had greater soil respiration and nitrogen, indicating increases in soil fertility. These rotations may offer an alternative to conventional crop-fallow rotations to counter ongoing declines in soil health.

**Supplementary Information:**

The online version contains supplementary material available at 10.1007/s00248-022-02074-w.

## Introduction

Microorganisms form the majority of soil biodiversity and underpin the provision of ecosystem services that support crop production, including soil organic matter (SOM) decomposition, nutrient cycling and aggregate formation [1, 2]. In dryland climates, conventional cropping systems typically have low crop rotational diversity and frequently implement fallow periods (periods where no plants are grown) to accumulate soil water for the next cash crop [3–5]. Low crop rotational diversity and frequent use of fallow periods have been demonstrated to have significant negative effects on soil microbial biomass [6, 7], resulting in negative impacts on microbially mediated ecosystem services and indicators of soil health [8–10]. To counter this, there is increasing interest in diversifying crop rotations and reducing the extent and frequency of fallow periods [3, 11, 12].

Cropping system diversification can be increased in several ways: (1) by increasing crop diversity per unit area and time, while maintaining the same cropping intensity (e.g. by converting from a wheat-fallow rotation to a wheat-fallow-chickpea-fallow rotation); (2) by intensifying the crop rotation to increase the number of crop harvests per unit area and time (e.g. double cropping); (3) by taking land out of annual crop production for a number of seasons and thereby extensifying the rotation (e.g. by introducing a ley pasture for two or more years before returning to annual cropping); or (4) by planting cover crops (i.e. non-cash crops) between cash crops to provide living ground cover.

Cropping system diversification can influence soil microbes due to differences in disturbance, and substrate quantity, quality, and availability compared with conventional cropping systems, as well as differences in inputs necessitated by growing different crops (e.g. different fertiliser regimes and herbicides). For example, increasing crop diversity enhances the diversity of crop residues, which differ in biochemical properties (e.g. C:N ratios and lignin content). This diversification of SOM could support greater niche diversity for soil microbes, leading to changes in soil microbial community diversity [13]. In addition, different plant species recruit specific microbial communities through their production of root exudates and signalling molecules [13, 14], which can have legacy effects for subsequent crops [15, 16]. These effects can differ between soil depths. Different crops within a rotation can vary in their root architecture, and as a result can influence different parts of the soil profile [17]. The upper layer of topsoil is also subject to greater variations in soil moisture and is more influenced by aboveground organic matter inputs than deeper soil layers [18, 19]. These factors can interact with rotation mediated effects on the soil microbiome. Extensifying rotations to include perennial crop phases can provide a more stable soil environment compared with annual crop-fallow rotations, due to reductions in tillage and the presence of continuous living plant cover. Reducing the frequency and intensity of soil disturbance in highly disturbed cropping systems promotes a greater abundance and diversity of disturbance-sensitive organisms, such as soil fungi [20, 21].

Across climate types, enhancing crop diversity can increase soil microbial biomass, influence microbial community composition, and less commonly, increase microbial species richness and evenness [8, 22, 23]. In semi-arid and sub-humid climates, intensifying dryland crop rotations to increase the number of crop harvests per unit time has been demonstrated to increase soil fungal biomass and fungal/bacterial ratios relative to conventional crop-fallow rotations [24], as well as soil metabolic diversity and microbial activity [25]. In a temperate oceanic climate (i.e. non-dryland climate), extensifying crop rotations with a multi-year grass ley pasture was found to promote greater biomass of bacteria and arbuscular mycorrhizal fungi (AMF), but to have little impact on microbial community composition [26]. In contrast, inclusion of lucerne (*Medicago sativa* L.) for 3 years in an annual crop rotation in a Mediterranean climate resulted in significant changes in soil microbial community composition [27]. Recent research in semi-arid North America found that soil microbial community size and fungal abundance were greater under cover crops compared with fallow, and that changes in soil microbial biomass and activity were dependent on both cover crop biomass production and species composition [6].

While cropping system diversification can influence soil microbial communities, most studies have focussed on non-dryland climates; hence, knowledge of these impacts in drylands is limited. Here, we used phylogenetic marker gene sequencing to compare soil bacterial and fungal alpha and beta diversity between a range of dryland conventional and diversified cropping systems in subtropical Australia. Conventional systems were cereal-dominant and followed a crop-fallow rotation. Diversified systems included those with greater crop rotational diversity, increased cropping intensity, inclusion of cover crops, and an extensified system with a multi-year ley pasture. Both summer and winter dominant crop rotations were included. We hypothesised that the diversified systems would result in significant shifts in soil microbial diversity and composition compared with the conventional systems. Furthermore, we hypothesised that these shifts would be associated with changes in soil properties related to cropping systems diversification, such as changes in SOM content, and would differ in magnitude with soil depth.

## Methods

### Study Site and Experimental Design

The study was conducted at the Northern Farming Systems experiment at Pampas, southeast Queensland, Australia (27° 44′ S, 151° 20′ E). The local climate is semi-arid [28, 29], with mean annual rainfall of 649 mm (30-year average) and an average annual temperature of 27.0 °C. The soil at the site is a black vertosol. The site has been cropped for over 50 years, with no-till management adopted more than 20 years ago. All experimental systems were managed under full stubble retention and sown using no-till planters. Baseline soil properties for the site are provided in Table 1.

**Table 1:** Baseline soil properties at the Northern Farming Systems experiment. Soils were sampled in Mar 2015, before the first experimental crops were sown

Soil properties	Shallow (0–10 cm)	Deep (10–30 cm)
TOC^a^ (g kg^−1^)	12.2	10.6
Colwell P (mg kg^−1^)	64.6	31.3
Colwell K (mg kg^−1^)	479	283
Bulk density (g cm^−3^)	1.24	1.24
pH (H_2_O)	7.4	8.1

^a^Walkley–Black method [30]

**Fig. 1:**
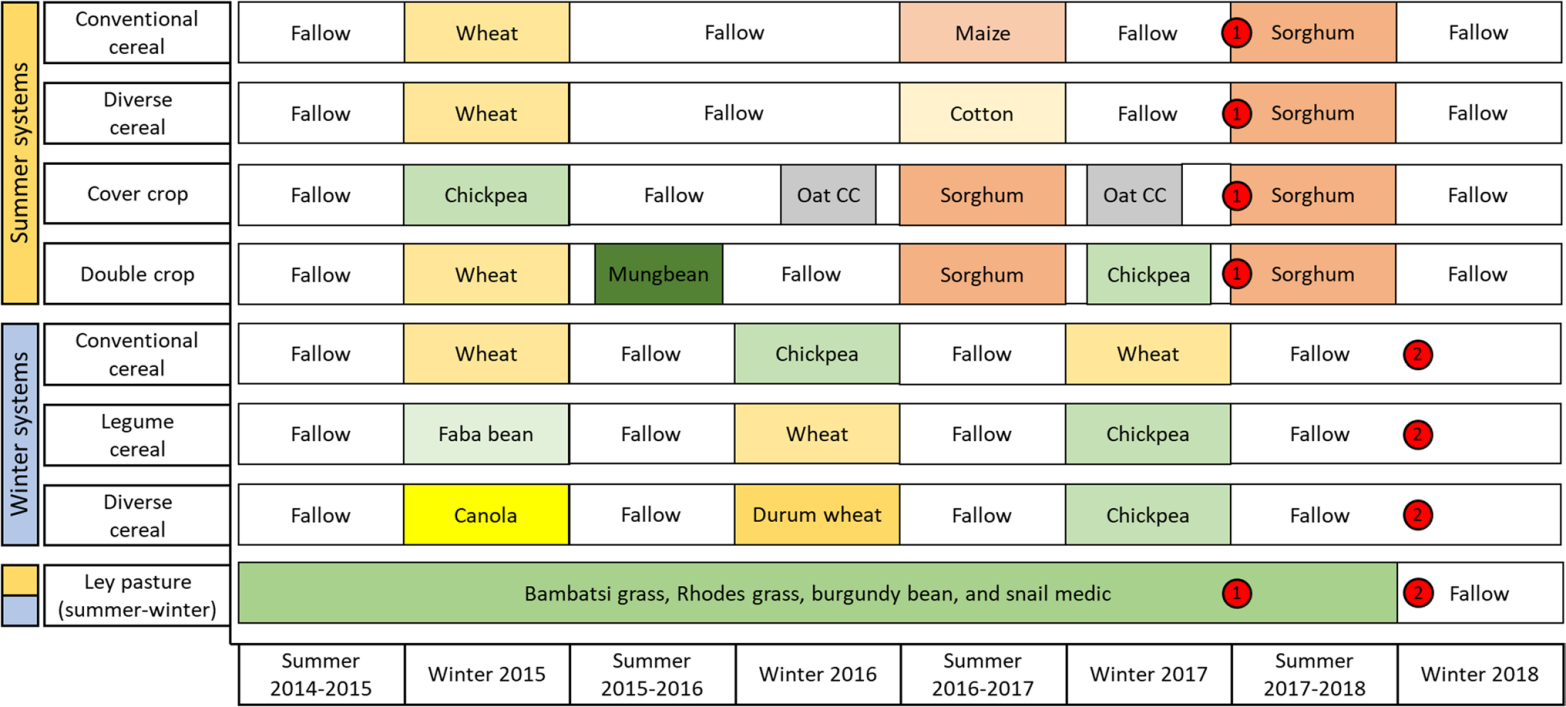
Timeline of crops grown in the sampled crop rotations between Dec 2014 and Aug 2018. Timings of soil sampling events in each rotation are shown with red dots: ‘1’ refers to summer season sampling; ‘2’ refers to winter season sampling. The Permanent Fallow is not shown in the diagram

The Northern Farming Systems experiment was established in March 2015 and includes 38 farming systems replicated within four blocks. The experiment is described in more detail in Bell et al. [31]. Of these 38 systems, we selected nine to investigate. The crop rotation history for these nine systems is shown in Fig. 1. Four of the systems were characterised by summer-dominant crop rotations; three were characterised by winter-dominant crop rotations; and two of the systems (Permanent Fallow and Ley Pasture) were consistent year-round. The summer cropping systems were comprised of the following: Conventional Cereal—a conventional cereal-dominant cropping system; Diverse Cereal—a more diverse cropping system that included non-cereal crops; Cover Crop—a system that incorporated cover crops; and Double Crop—an intensified system that included double cropping. The winter cropping systems were comprised of the following: Conventional cereal—a conventional wheat-chickpea system; Legume Cereal—a diversified cropping system that included greater use of legume crops; and Diverse Cereal—a second diversified cropping system that included a variety of cereal and non-cereal crops (Fig. 1). The Ley Pasture was planted with a 25:25:25:25 (g/g) mixture of Bambatsi grass (*Panicum coloratum*, L.), Rhodes grass (*Chloris gayana*, Kunth), burgundy bean (*Macroptilium bracteatum*, Nees & Mart.) and snail medic (*Medicago scutellate*, (L.) Mill.). The Ley Pasture was terminated with glyphosate in May 2018 in preparation for return to grain cropping. The Permanent Fallow had no crops planted and was kept weed-free by using contact herbicides for the duration of the experimental period. For further information on how the cropping systems were managed, see Williams et al. [32].

### Soil Sampling

Our intention was to examine the effect of cropping system history on soil microbial diversity, rather than differences between specific crops at a given time. To achieve this, systems were sampled when the rotation phase was most similar to minimise the impact of the current crop. As this could not be achieved across both summer and winter cropping systems, these were sampled and analysed separately. Soil sampling was completed in two events: summer season sampling (for the summer cropping systems) and winter season sampling (for the winter cropping systems). The summer cropping systems, Ley Pasture, and Permanent Fallow were sampled on 14 Nov 2017 (summer season sampling). This coincided with a sorghum (*Sorghum bicolor* (L.) Moench) phase in the Conventional Cereal, Diverse Cereal, Cover crop and Double Crop systems. The winter cropping systems, Ley Pasture and Permanent Fallow were sampled on 11 Jun 2018 (winter season sampling). This coincided with a fallow period across the Conventional Cereal, Legume Cereal and Diverse Cereal systems; these systems were due to be planted to wheat (*Triticum aestivum* L.) but were left fallow due to drought.

At both sampling events, twelve soil cores (34 mm diameter) were collected at two depths (0–10 cm [shallow] and 10–30 cm [deep]) from each plot (four plots per rotation). The twelve cores per depth per plot were combined to create a composite plot sample at each depth. Soil cores were collected at random in the Ley Pasture and Permanent Fallow plots and were taken from crop inter-rows in all other plots. Samples were stored in sealed plastic bags on ice while in transit to the laboratory to minimise changes to the soil microbial community before DNA extraction. All samples were minimally disturbed before being passed through a 10 mm sieve and then frozen at − 20 °C. Soils were kept frozen until DNA extraction.

### Soil Abiotic Parameters and Respiration

The samples collected in the summer season were analysed for mineral nitrogen (N) (sum of NO_3_ + NH_4_) via 2 M KCl extraction [33] and soil basal respiration after incubation for 24 h at 22 °C [34]. In addition, the samples were fractionated based on particle size to determine total organic C and N (TOC and TON, respectively), particulate organic C and N (POC and PON, respectively), and mineral-associated C and N (MAOC and MAON, respectively)[35, 36]. Full descriptions of these analyses are provided in Williams et al. [32]. The samples collected in the winter season were only analysed for mineral N and soil basal respiration due to budgetary constraints.

### Phylogenetic Marker Gene Sequencing

DNA was extracted from 250 mg (fresh weight) of each soil sample in triplicate using the DNeasy PowerSoil kit (Qiagen) according to the manufacturer’s instructions. The DNA from the technical replicates were pooled; these pools were used as biological replicates in subsequent analysis.

To characterise the diversity of soil fungal communities, ITS2 genes were polymerase chain reaction (PCR) amplified using the primers gITS7 (5′-GTG AAT CAT CGA ATC TTT G-3′) [37] and ITS4 (5′-TCC TCC GCT TAT TGA TAT GC-3′) [38]. To assess the diversity of soil bacterial communities, 16S rRNA genes were PCR amplified using the primer pair 926F (5′-AAA CTY AAA KGA ATT GRC GG-3′) and 1392wR (5′-ACG GGC GGT GWG TRC-3′). The 5′ end of all primers was modified to contain the Illumina overhang adapter for compatibility with the P5 and i7 Nextera XT indices. PCRs contained 2 µL of DNA template combined with 4 µL 5X Phire Green Reaction Buffer (Thermo Fisher), 100 µM of each dNTP (Invitrogen), 0.4 µL Phire Green Hot Start II DNA Polymerase (Thermo Fisher), 10 mM of each primer. This mixture was made up to a total volume of 20 µL with molecular biology grade water. Thermocycling conditions were the same for all reactions: 98 °C for 45 s; then 35 cycles of 98 °C for 5 s, 56 °C for 5 s, 72 °C for 6 s, followed by 72 °C for 1 min. Amplifications were performed using a SimpliAmp 96-well Thermocycler (Applied Biosystems).

Magnetic beads were used to purify amplicons from the PCRs [39] and these were dual indexed using the Nextera XT Index Kit (Illumina) according to the manufacturer’s instructions. Indexed amplicons were purified using magnetic beads and then quantified using a PicoGreen dsDNA Quantification Kit (Invitrogen). Equimolar concentrations of each sample were pooled and sequenced on an Illumina MiSeq using 30% PhiX Control v3 (Illumina) and a MiSeq Reagent Kit v3 (600 cycles, Illumina) according to the manufacturer’s instructions.

Sequence data were processed using a modified UPARSE approach [40, 41]. Unique barcodes on each sample were used to demultiplex pools using the cutadapt tool in QIIME2 [42]. ITS2 or 16S sequences then followed a separate bioinformatics pipeline. For 16S sequences, fastx_truncate of USEARCH was used to remove primer trim sequences to 250 bases [43]. Fastq_filter of USEARCH was used to filter trimmed reads with a maxee score > 1.0. For ITS2 sequences, ITSx with fungi as the specified profile was used to extract ITS2 sequences [44]. Chimeras were removed from extracted sequences using uchime2_ref of USEARCH against the UNITE 8.2 database [45]. ITS2 and 16S sequences then resumed with the same pipeline after these steps had completed. Fastx_uniques and cluster_otus of USEARCH were used to generate representative sequences of sequences with a similarity of > 97%. Reads were mapped against these representative sequences to create an operational taxonomic unit (OTU) table using the otutab function in USEARCH. Taxonomy was assigned from the SILVA 128 [46] database for 16S sequences and UNITE 8.2 database for ITS2 sequences using blantn from QIIME2. OTUs not assigned as bacteria or fungi were filtered out from further analyses. 16S representative sequences were aligned using MAFFT (v7.221) [47] and masked using QIIME2 to generate phylogenetic distance. From this alignment, a midpoint-rooted phylogenetic tree was generated using FastTree (v2.1.9, [48]. 16S and ITS2 reads were rarefied to 2100 and 3500 per sample, respectively. Alpha diversity metrics were calculated using the *qiime diversity alpha-phylogenetic* and *qiime diversity alpha* functions from QIIME2.

### Statistical Analysis

Differences in community composition were assessed using PERMANOVA and redundancy analysis using R package *vegan *[49]*.* Significant (*P* ≤ 0.05) correlations of community composition with abiotic variables were assessed using the envfit function in *vegan*. In addition, community composition was assessed using multivariate generalised linear models (GLMs) assuming a negative binomial distribution, implemented through the *mvabund* package [50]. Sum-of-likelihood tests to compare experimental models to alternative models created via resampling were conducted to determine significant differences, including *post hoc* comparisons [51]. Benjamini–Hochberg corrections for multiple comparisons were applied to multivariate GLM *post hoc* analyses using base R. Alpha diversity metrics were assessed through ANOVA tests of linear models with sampling depth and cropping system as main effects. *Post hoc* analyses from univariate models was conducted using estimated marginal means with Benjamini–Hochberg corrections using the R package *emmeans* [52]. Species that were indicative of changes in community composition were identified using the indval function from the *labdsv* package [53]. Indicator species shown were filtered from all indicator species to those that, within at least one cropping system, had more than ten reads on average.

Differences in abiotic data for summer-dominant rotations between groups were assessed using PERMANOVA and the estimated marginal means of a multivariate linear model produced using *mvabund*. Abiotic variables from winter-dominant rotations were assessed using univariate linear models and *post hoc* analysis was conducted using estimated marginal means with Benjamini–Hochberg corrections.

## Results

### Soil Abiotic Parameters

#### Summer Season

**Fig. 2:**
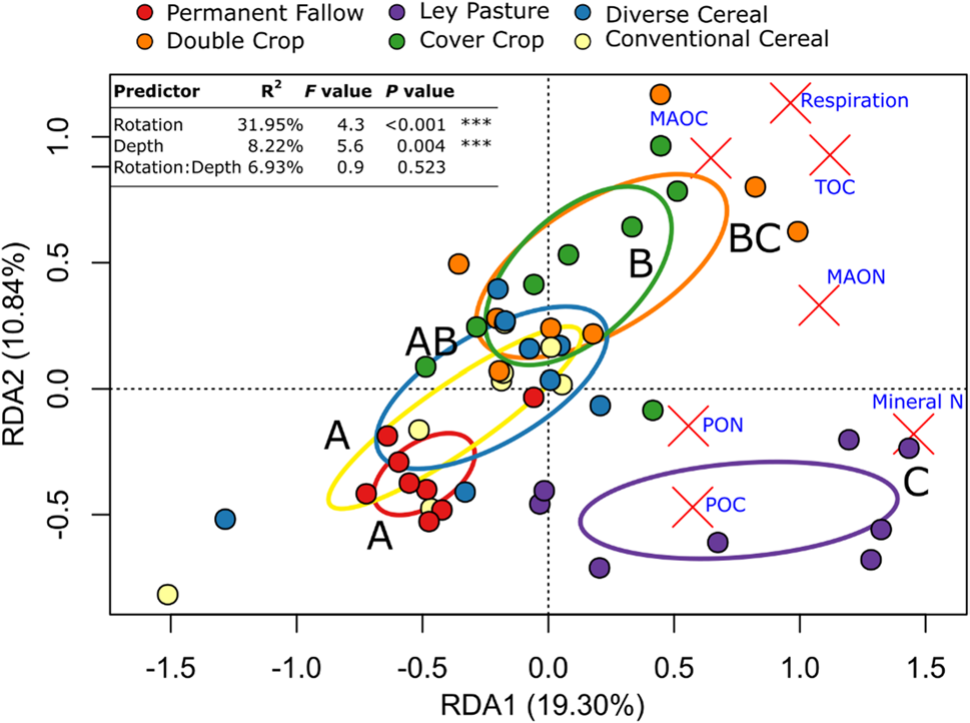
Redundancy analysis of z score transformed abiotic soil variables from different summer broadacre rotation systems. Ellipses represent standard deviations of the group centroid. Letters represent different groups according to estimated marginal means with Benjamini–Hochberg corrections from a multivariate linear model of the different groups shown. Results from a PERMANOVA test are inset. Abbreviations: total organic carbon (TOC), mineral associated organic nitrogen (MAON), mineral associated organic carbon (MAOC), particulate organic nitrogen (PON), and particulate organic carbon (POC)

Soil abiotic variables differed significantly between soil depths and crop rotations (Fig. 2). No significant depth × rotation interactions were observed. Relative to other rotations, the abiotic properties associated with Ley Pasture were the most distinct and were characterised by larger mineral N, PON and POC concentrations (Fig. 2). Conversely, the Conventional Cereal rotation and Permanent Fallow were negatively associated with the measured C and N fractions (Fig. 2).

#### Winter Season

More mineral N content was observed at Shallow than Deep sampling depths (Table 2, Fig. S1). Rotation also influenced mineral N; mineral N was 40–63% higher on average in Ley Pasture relative to other rotations (Table 2, Fig. S1). No interaction was observed between depth and rotation on mineral N. Depth had the largest effect on soil respiration with respiration generally being greater in Shallow than Deep sampling depths (Table 2). A main effect of rotation was also observed. However, there was an interaction effect between depth and rotation; respiration was only significantly different between depths for the Diverse Cereal and Ley Pasture (Table 2, Fig. S2).

**Table 2:** Results from ANOVA tests to assess the impact of winter broadacre rotation and sampling depth soil abiotic variables

Variable	Predictor	*F* value	*P* value
Mineral N	Rotation	18.7	< 0.001***
	Depth	7.4	0.011*
	Rotation/depth	1.6	0.195
Soil respiration	Rotation	3.5	0.019*
	Depth	21.8	< 0.001***
	Rotation/depth	3.7	0.015*

### Soil Microbial Communities

#### Alpha Diversity

A main effect of depth on the number of observed bacterial and fungal OTUs was detected for both summer and winter cropping rotations (Tables 3 and 4). In summer systems, more bacterial and fungal OTUs were observed at Deep depth; however, in winter systems, more bacterial and fungal OTUs were observed at Shallow depth (Fig. S3). According to Faith’s PD, depth did not influence phylogenetic diversity in measured bacterial communities (Table S1). When evenness was also considered (Shannon diversity), only bacteria in summer systems were affected by sampling depth (Tables S1 and S2).

**Table 3:** Results from linear models and multivariate tests to assess the impact of broadacre rotation and sampling depth on the alpha and beta diversity of soil bacterial communities

Season	Predictor	Observed OTUs (alpha diversity)		PERMANOVA (Hellinger) (beta diversity)			Multi-GLM (beta diversity)
		*F* value	*P* value	*R*^2^	*F* value	*P* value	*P* value
Summer	Rotation	2.6	0.043*	13.12%	1.3	0.002**	0.005**
	Depth	4.7	0.037*	4.79%	2.3	< 0.001***	0.005**
	Rotation:depth	0.9	0.490	9.06%	0.9	0.981	0.770
Winter	Rotation	0.4	0.772	11.06%	1.2	0.099	0.015*
	Depth	10.0	0.004**	13.33%	5.6	< 0.001***	0.005**
	Rotation:depth	0.8	0.545	8.63%	0.9	0.787	0.465

**Table 4:** Results from linear models and multivariate tests to assess the impact of broadacre rotation and sampling depth on the alpha and beta diversity of soil fungal communities

Season	Predictor	Observed OTUs (alpha diversity)		PERMANOVA (Hellinger) (beta diversity)			Multi-GLM (beta diversity)
		*F* value	*P* value	*R*^2^	*F* value	*P* value	*P* value
Summer	Rotation	3.2	0.017*	21.00%	2.4	< 0.001***	0.005**
	Depth	4.6	0.039*	6.42%	3.6	< 0.001***	0.005**
	Rotation:depth	0.5	0.779	8.46%	1.0	0.670	0.005**
Winter	Rotation	4.6	0.005**	18.89%	2.1	< 0.001***	0.005**
	Depth	111.3	< 0.001***	5.54%	2.5	< 0.001***	0.005**
	Rotation:depth	1.4	0.256	8.14%	0.9	0.867	0.005**

**Fig. 3:**
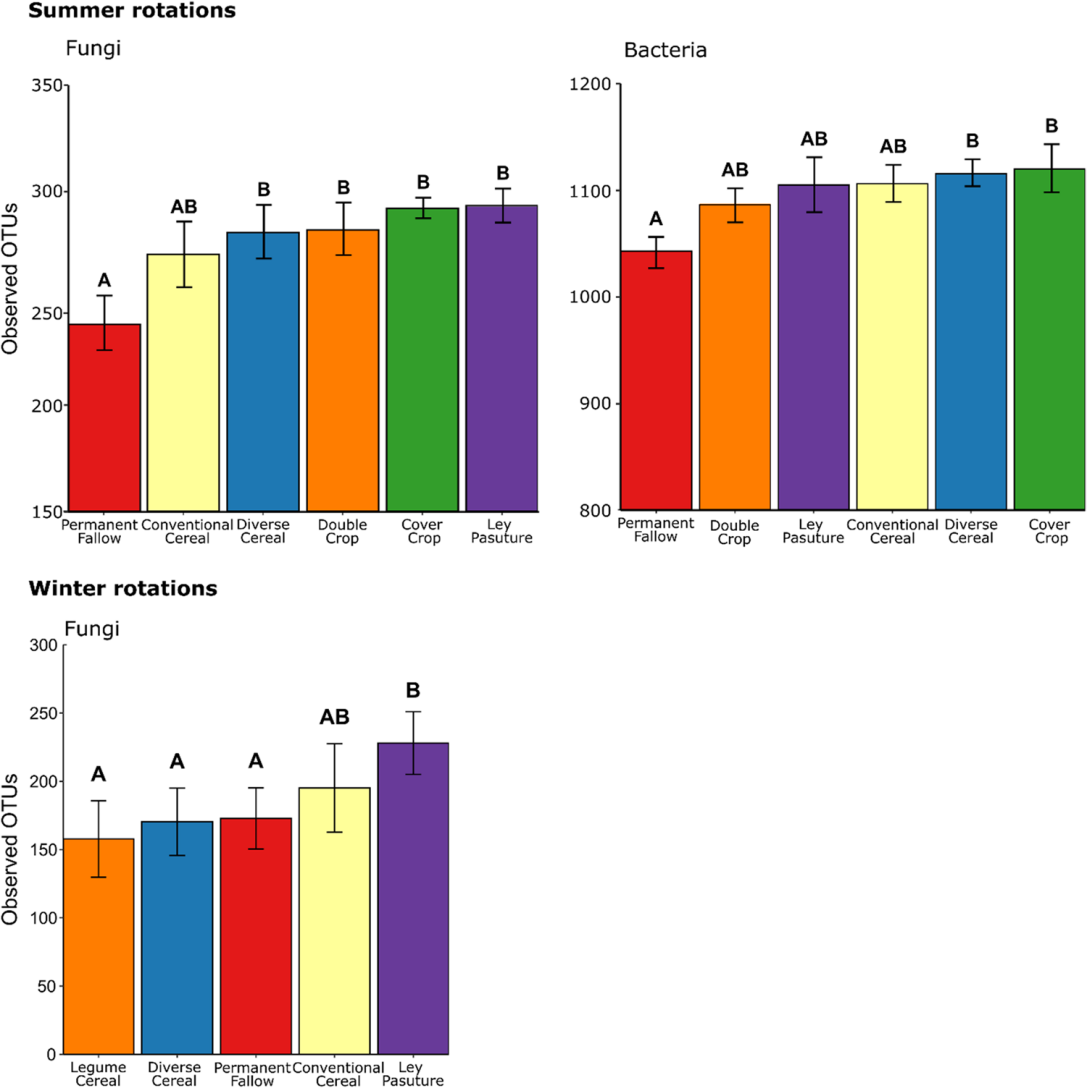
Bar charts showing the mean number of observed bacterial and fungal OTUs in different summer and winter broadacre rotations. Letters indicate a significant difference in observed bacterial or fungal OTUs within either summer or winter broadacre rotations according to *post hoc* analysis with Benjamini–Hochberg corrections. Error bars represent standard errors

Winter cropping rotations had no significant effects on bacterial alpha diversity according to any metric (Tables 3 and S1), but they did have a main effect on the number of observed and predicted fungal OTUs (Tables 4 and S2). Within the winter cropping rotations, Ley Pasture had significantly more observed fungal OTUs that all other systems except for the Conventional Cereal system (Fig. 3). Summer cropping rotations had significant effects on both bacterial and fungal alpha diversity according to multiple metrics (Tables 3, 4, S1 and S2). Within the summer rotation, the Diverse Cereal and Cover Crop rotations increased both bacterial and fungal observed OTUs compared to the Permanent Fallow (Fig. 3). Ley Pasture and the Double Crop rotation also increased fungal observed OTUs compared to the Permanent Fallow but not the number of bacterial observed OTUs (Fig. 3). No interaction effects between rotation and depth were observed for bacteria or fungi according to any alpha diversity metric (Tables 3, 4, S1 and S2).

#### Beta Diversity

##### Summer Rotations

Depth and the summer cropping rotations significantly influenced bacterial and fungal community composition (Table 3 and 4). There was no interaction between these factors according to PERMANOVA tests. However, multivariate GLMs detected an interaction between depth and rotation on the composition of fungal communities (Table 4).

**Fig. 4:**
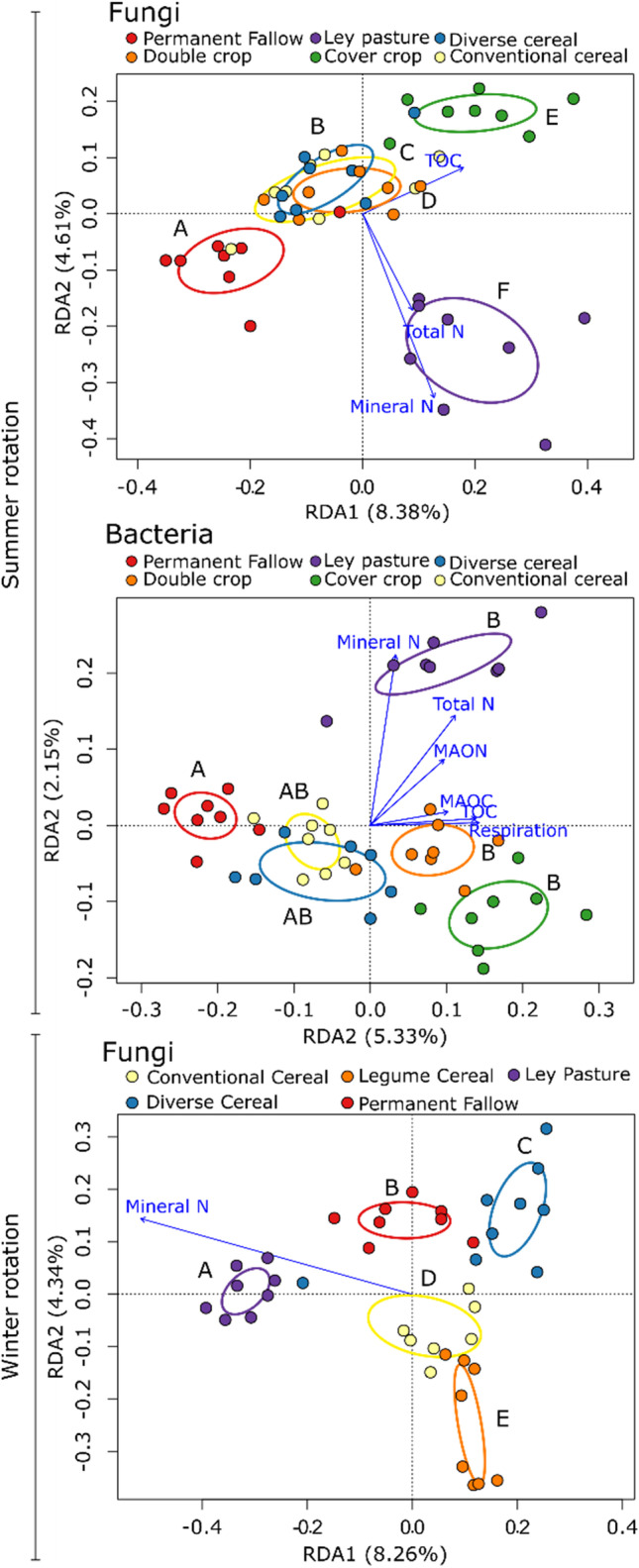
Redundancy analysis of bacterial and fungal OTUs in different summer and winter broadacre rotations. Letters indicate a significant difference in composition within either summer or winter broadacre rotations according to GLM *post hoc* analysis with Benjamini–Hochberg corrections. Ellipses represent standard deviations of centroids. Arrows represent significant correlations of community composition with abiotic variables, lengths of the arrows represent the strength of the correlation

The Ley Pasture fungal community was significantly correlated with the TON and mineral N present in the soil (Fig. 4). Multiple OTUs from the fungal order Pleosporales were positively associated with the Ley Pasture (Fig. S4). In contrast, the fungal community in the Cover Crop rotation was correlated with TOC (Fig. 4). The Cover Crop and Ley Pasture rotations had the most fungal indicator species (15 each, Fig. S4). The Shallow depth fungal community was associated with greater concentrations of SOM (TOC, TON and MAON), and higher rates of respiration (Fig. S5). Forty-eight OTUs were indicative of a specific sampling depth (Fig. S6). The most abundant OTU associated with Shallow depths was a *Fusarium* sp. (OTU 2), while the most abundant OTUs associated with Deep depths were *Alternaria* sp. (OTU 5).

The summer rotations had a less pronounced effect on bacterial community composition: Permanent Fallow soils were distinct from Ley Pasture, Double Crop and Cover Crop soils, which were in turn correlated with higher soil C, N and basal respiration (Fig. 4). Permanent Fallow soils also had the greatest number of bacterial indicator OTUs (8) which included members of the Gaiellales, Rubrobacterales, Solirubrobacterales, and a single representative from the Gemmatimonadetes (Fig. S7). Similar to fungal communities, Shallow depth bacterial communities were associated with more C, N and respiration (Fig. S5). OTU 1 (*RB41* sp.) was the most abundant bacterial indicator of the Shallow depth (Fig. S8). OTU 17 a member of the order Acidimicrobiales, was the most abundant bacterial indicator of the Deep depth (Fig. S8). Thirty-one bacterial OTUs were indicative of sampling depth in the summer rotations (Fig. S8).

##### Winter Rotations

Depth significantly influenced bacterial and fungal community composition within winter cropping rotations (Tables 3 and 4). Fungal community composition was different between the winter cropping rotations, but not bacterial community composition (Tables 3 and 4). Multivariate GLMs, but not PERMANOVA, detected an interaction between depth and rotation on fungal communities (Table 4).

All fungal communities arising from the different cropping rotations were distinct (Fig. 3). Again, Ley Pasture community composition was correlated with an increase in mineral N (Fig. 4). Fifty-two percent of indicator fungal OTUs (32) were associated with Ley Pasture, the most of any rotation (Fig. S9). Indicators of Ley Pasture were phylogenetically diverse and included representatives of the orders Pleosporales, Hypocreales and Sordariales (Fig. S9). Fungal communities associated with the Shallow sampling depth were only associated with increased soil respiration (Fig. S5). Forty-seven fungal OTUs were indicative of sampling depth; > 89% (42) of these were associated with the Shallow sampling depth (Fig. S10). The majority of these were Ascomycota (40) were associated with depth, 38 of these were associated with Shallow sampling depths (Fig. S10).

Bacterial communities from the Shallow sampling depth were associated with higher rates of soil respiration, C and N (Fig. S5). Ninety bacterial OTUs were indicative of sampling depth, the most of any treatment, and came from eight different phyla (Figs. S11 and S12).

## Discussion

In dryland climates, fallow periods have been implemented as a method of accumulating soil moisture between crop phases [3–5]. However, fallowing has been shown to have significant impacts on soil microbes [6, 7, 54], which provide critically important ecosystem services including SOM turnover and soil structure development [8–10]. Here, we have shown that diversified dryland cropping rotations impact soil microbial communities and that these impacts correlate with abiotic changes in the soil environment.

We observed a change in fungal, but not bacterial community composition and richness between winter rotations. Previous research has shown that changes in organic matter inputs can lead to rapid, but ephemeral changes in copiotrophic microbes—these microbes utilise labile C compounds for energy and growth [55]. This period can be followed by the succession of more persistent changes in fungal communities that are able to decompose the more recalcitrant C compounds that remain [56, 57]. This may explain the absence of any observable change in bacterial community composition and richness between winter rotations, where drought prevented planting of new crops and enforced an extended fallow period. This would have limited new labile C inputs into the soil prior to sampling. In contrast, at the point of sampling the summer rotations were unaffected by drought, meaning there were ongoing organic matter inputs from the presence of crops. These fresh inputs may have supported the continued presence of copiotrophs, resulting in observable changes in bacterial and fungal OTU richness and community composition between the summer rotations.

Fallow periods with minimal crop residue to cover soil and that are maintained weed-free are known to have significant impacts on soil microbial communities[6, 7, 54]. The Permanent Fallow, which had no living plant cover (i.e., was maintained weed-free with herbicides) or other inputs of organic matter for 3 years prior to the summer-season sampling, was among the rotations that supported the fewest bacterial and fungal OTUs (Fig. 3). This likely occurred because the absence of fresh organic matter inputs via rhizodeposits and crop residues led to decreasing nutrient resources. As a result, there were fewer species that could survive this low nutrient environment [58, 59] and the microbial community became increasingly dominated by fewer species that could decompose recalcitrant organic matter [56, 60]. This is important as losses in soil microbiome diversity can lead to reductions in ecosystem functions that are important to plant health [61].

We hypothesised the diversified cropping rotations would support greater fungal richness than the conventional cropping rotations. This would have possibly occurred due to a greater diversity of residue inputs opening more functional niches [62], but this did not happen. It is feasible that the quantity and quality of the root exudates and crop residues produced in the summer crop rotations were insufficiently diverse to generate significant shifts in fungal richness, or that more time is needed to produce such shifts [7, 63]. In the winter crop rotations, fungal OTU richness was greatest in the Ley Pasture, while the Conventional Cereal and Diverse Cereal rotations supported similar numbers of fungal OTUs as the Permanent Fallow. Ongoing drought led to the implementation of long fallows (> 6 months) across all the cropping rotations except the Ley Pasture. This reduction in nutrient inputs also likely led to a reduction in fungal OTU richness; however, the Ley Pasture, which supported greater fungal richness, maintained living plant cover for longer. This continued plant biomass may have also sustained more fungal taxa, resulting in greater richness.

Cropping rotation diversification led to significant shifts in soil microbial community composition across both summer and winter crop rotations. In all cases, the Permanent Fallow had the most distinct communities. This is likely related to the C and N resource constraints described earlier, shifting community composition towards greater dominance of saprophytic taxa able access highly recalcitrant nutrient forms or survive low nutrient conditions. This is exemplified by the identification of several actinobacteria OTUs as indicator taxa for the Permanent Fallow—actinobacteria have been shown to adapt to the warm and dry conditions that are typical of Australian soils [64]. The Ley Pasture also developed distinct bacterial and fungal communities, with clear separation based on soil TON and mineral N concentrations. The accumulation of N in the Ley Pasture is likely based on the maintenance of perennial plant cover that included 50% N-fixing legumes. Greater incorporation of legumes into crop rotations is considered an important tool in reversing soil fertility decline in agricultural systems by building soil C and N stocks [8, 65]. Legume incorporation into crop rotations has been demonstrated to result in shifts in soil organic N levels over relatively short time scales (< 3 years [32]). The Cover Crop rotation also supported distinct soil microbial communities. In contrast to the Ley Pasture, the Cover Crop rotation’s community composition separated from other rotations based on increases in soil C rather than N. Given the similarity in the quantities of plant biomass produced in the Cover Crop rotation and Ley Pasture between 2015 and 2019 [32], it is likely that the different plant species compositions of these rotations (Fig. 1) led to recruitment of distinct soil microbial communities [14, 59]. The cereal-dominant nature of the plant cover in the Cover Crop rotation could have resulted in greater inputs of higher C:N ratio residues compared with the Ley Pasture, which consisted of 50% legumes [66].

Within the summer cropping rotations, the Conventional Cereal and Diverse Cereal rotations showed a degree of similarity in soil microbial community composition. Due to the relatively short timescale since the establishment of the field experiment, the only difference between the summer Conventional Cereal and Diverse Cereal rotations was a maize versus cotton crop in the 2016–2017 season. It is likely that this small degree of difference has not been sufficient to cause divergence in soil microbial community composition. In contrast, the Double Crop rotation had a greater degree of crop diversity (four crop species, including two legume species) compared with the Conventional Cereal rotation and showed evidence of divergent soil bacterial communities (Fig. 4). Shifts in bacterial community composition have been previously observed in rotations with increased use of legumes [22]. In addition, shifts in fungal community composition due to increased use of legumes have also been observed, namely a reduction in fungal diversity and an increase in fungal pathogens [22, 67]; however, such shifts were not observed in summer rotations of the current experiment. In contrast, distinct fungal communities were observed between all winter cropping rotations. It is not clear why such separation was observed during a long fallow, but it may be related to shifts in microbial composition away from copiotrophs and towards fungal taxa that are able to decompose more recalcitrant organic matter in the absence of fresh inputs of crop residues [56, 60].

Differences in soil microbial alpha diversity were observed across the two sampled soil depths. In the summer sampling, the number of observed bacterial and fungal OTUs were lower at 0–10 cm depth (Shallow) compared with 10–30 cm depth (Deep), but in the winter sampling they were greater at 0–10 cm depth compared with 10–30 cm depth. Differences in soil microbial communities across soil depth profiles has been observed across both natural and agricultural environments, driven by changes in factors such as soil aeration, moisture, temperature and nutrient availability [68, 69]. Changes in soil microbial community structure and functioning as a function of season have also been observed but without consistent trends [68]. It is possible that in the summer sampling higher soil temperatures combined with lower soil moisture caused microbial taxa to migrate deeper into the soil, causing fewer OTUs to be identified at this time in the 0–10 cm depth layer. In the winter sampling, there was a doubling of observed fungal OTUs at 0–10 cm depth compared with 10–30 cm depth. This is potentially due to a proliferation of saprophytic fungi in these rotations actively decomposing crop residues near the soil surface, as the ongoing drought meant no crops were planted and thus there were no fresh organic matter inputs at deeper soil depths.

## Conclusions

Cropping rotation diversification can lead to significant shifts in soil microbial community diversity and composition, especially when compared to soils under long-term fallow. Nevertheless, cover cropping and incorporating multi-year ley pastures generated microbial communities that were distinct from those in conventional cropping rotations. Moreover, the microbial communities within Cover Crop and Ley Pasture rotations were associated with increased soil organic C and N, pointing to improvements in soil fertility parameters in conjunction with microbial community turnover. These rotations therefore may be a preferable alternative to fallow periods that are often implemented in dryland cropping. Further research is needed to determine the implications of shifts in soil microbial community diversity and composition in terms of soil functional processes related to crop productivity.

## Supplementary Information

Below is the link to the electronic supplementary material.

Supplementary file1 (DOCX 2772 KB)

## Data Availability

The 16S and ITS rRNA gene amplicon sequences associated with this study have been deposited in the NCBI SRA under the BioProject accession: PRJNA819419. Soil abiotic data is stored in The University of Queensland UQ eSpace data repository (https://doi.org/10.14264/e1f6e2b) and is available on reasonable request.
