# Supplementary material for: Cropping System Diversification Influences Soil Microbial Diversity in Subtropical Dryland Farming Systems
Source: Microb Ecol. 2022 Jul 15;85(4):1473–84. doi: 10.1007/s00248-022-02074-w (PMC10167104; doi:10.1007/s00248-022-02074-w)
Supplement: Supplementary file 1 — Supplementary file1 (DOCX 2772 KB) [file 248_2022_2074_MOESM1_ESM.docx]

**
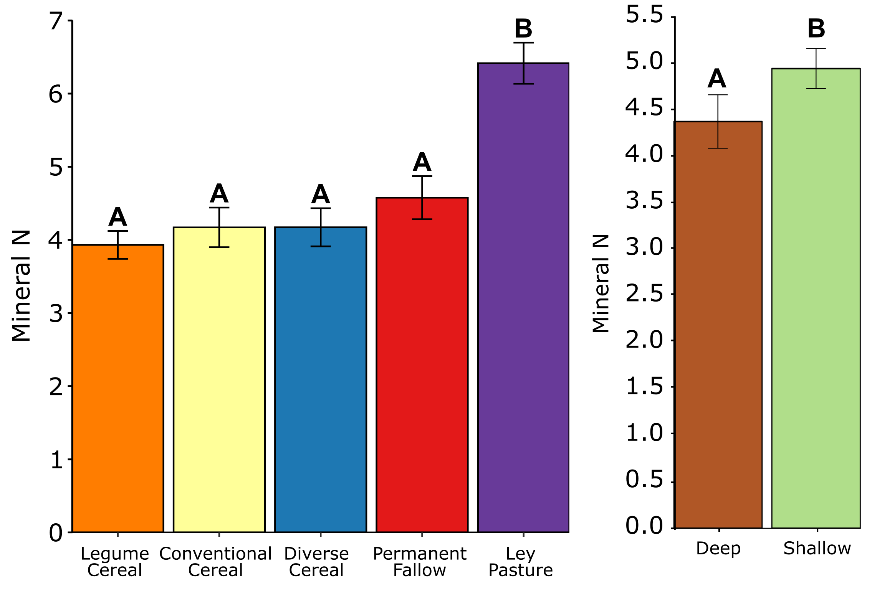
**

**Fig. S1** Bar charts showing the mean amount of soil mineral N between different sampling depths (0-10 cm [Shallow], 10-30 cm [Deep]) and winter rotation systems. Letters represent statistically different groups according to post hoc analysis using estimated marginal means with Benjamini-Hochberg corrections.

**
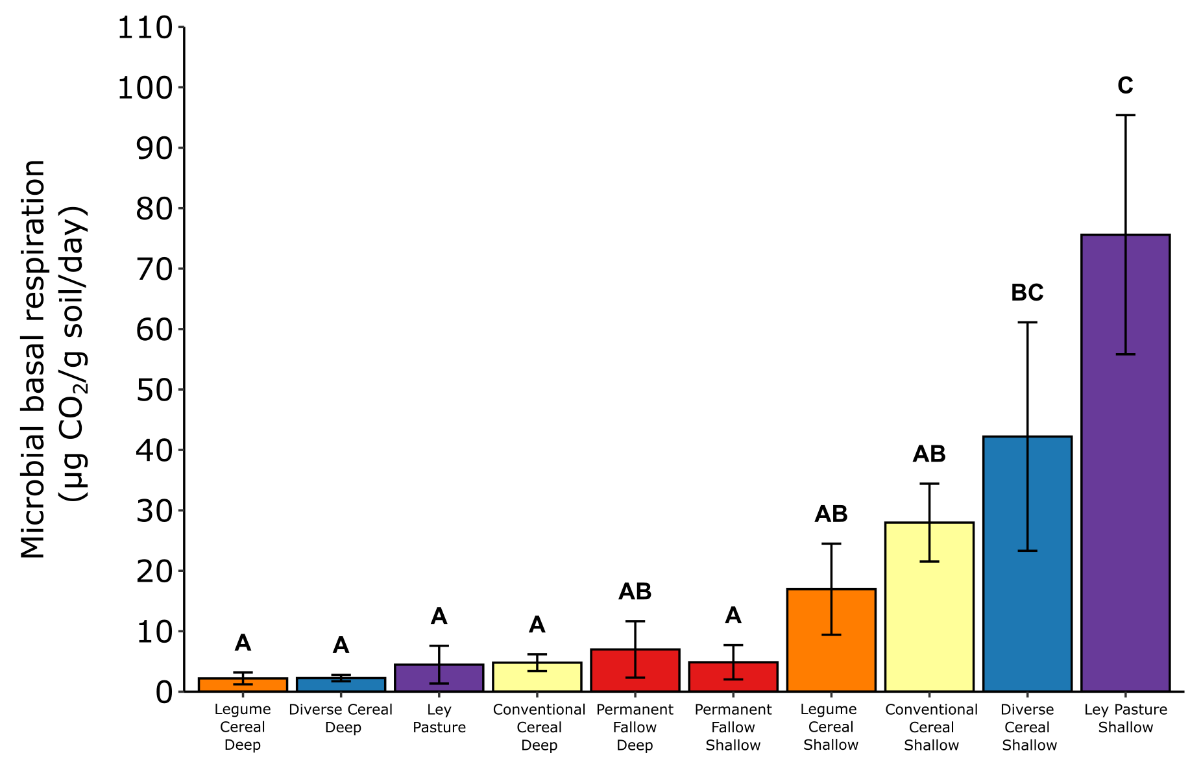
**

**Fig. S2** Bar charts showing the mean soil respiration between the interaction of different sampling depths (0-10 cm [Shallow], 10-30 cm [Deep]) and winter rotation systems. Letters represent statistically different groups according to post hoc analysis using estimated marginal means with Benjamini-Hochberg corrections.


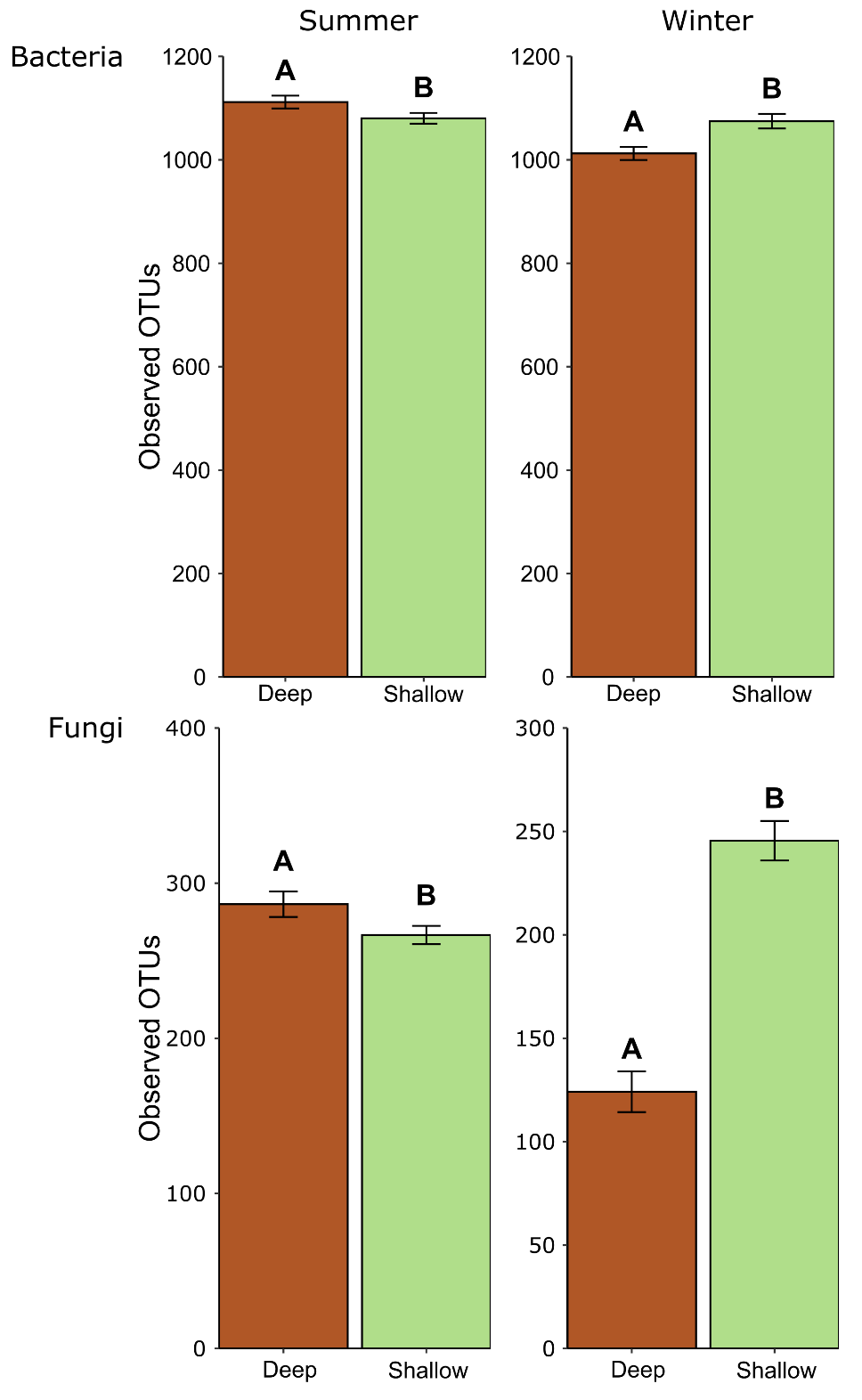


**Fig. S3** Bar charts showing the mean number of observed bacterial and fungal OTUs at different sampling depths (0-10 cm [Shallow], 10-30 cm [Deep]) in summer and winter broadacre rotations. Letters indicate a significant difference in observed bacterial or fungal OTUs within either summer or winter broadacre rotations. Error bars represent standard errors.

**Table S1** ANOVA results from linear models to assess the impact of sampling depth and broadacre rotation on soil bacterial alpha diversity metrics.

**Table S2** ANOVA results from linear models to assess the impact of sampling depth and broadacre rotation on soil fungal alpha diversity metrics.

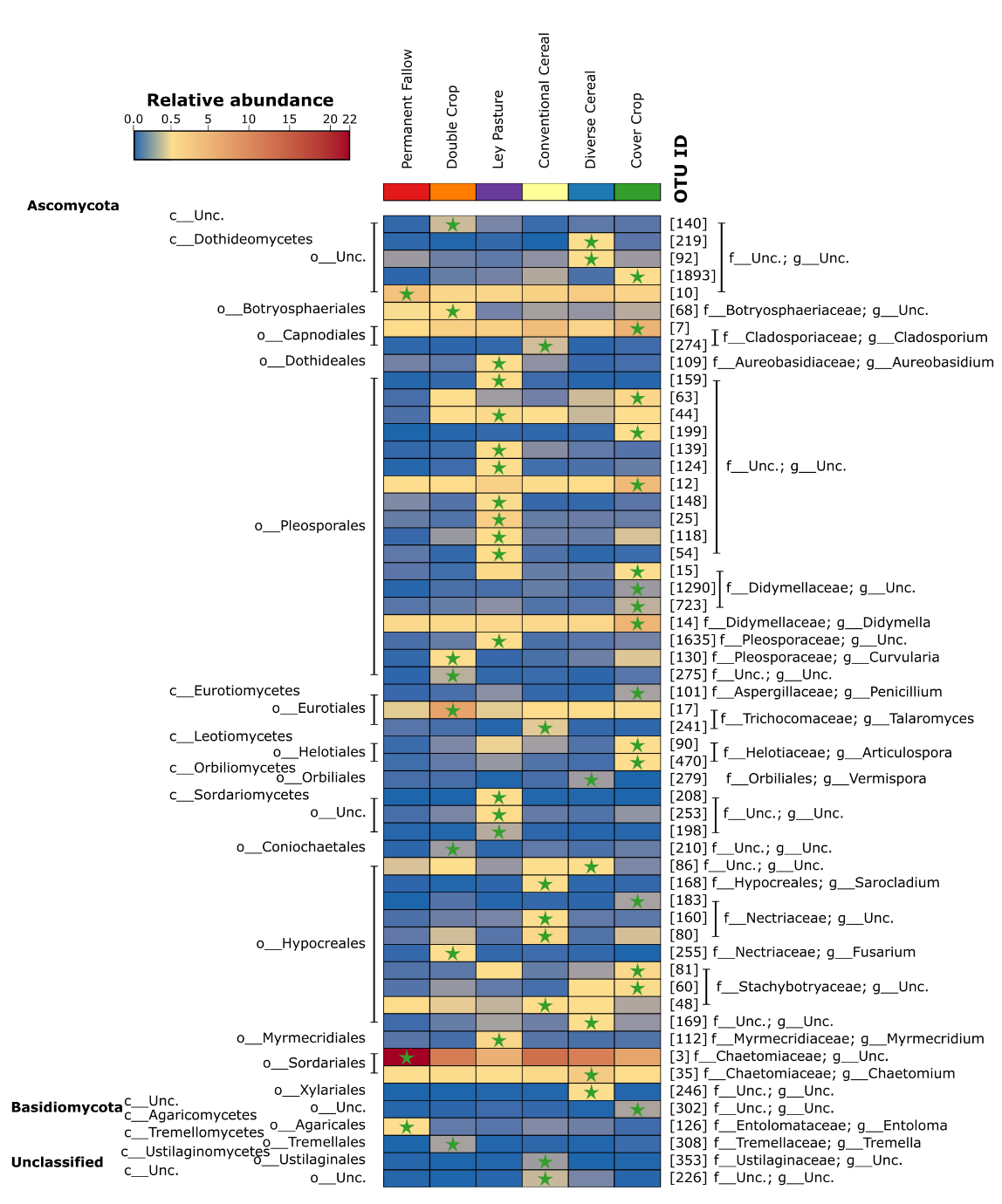


**Fig S4** A heatmap showing fungal indicator OTUs from different summer crop rotations. Tiles indicate mean relative abundances of OTUs within a rotation. Relative abundances between 0-0.5% are represented by a blue-yellow scale, those 0.5%-22% are represented by a yellow-red scale. Green stars indicate which rotation each OTU is an indicator of.


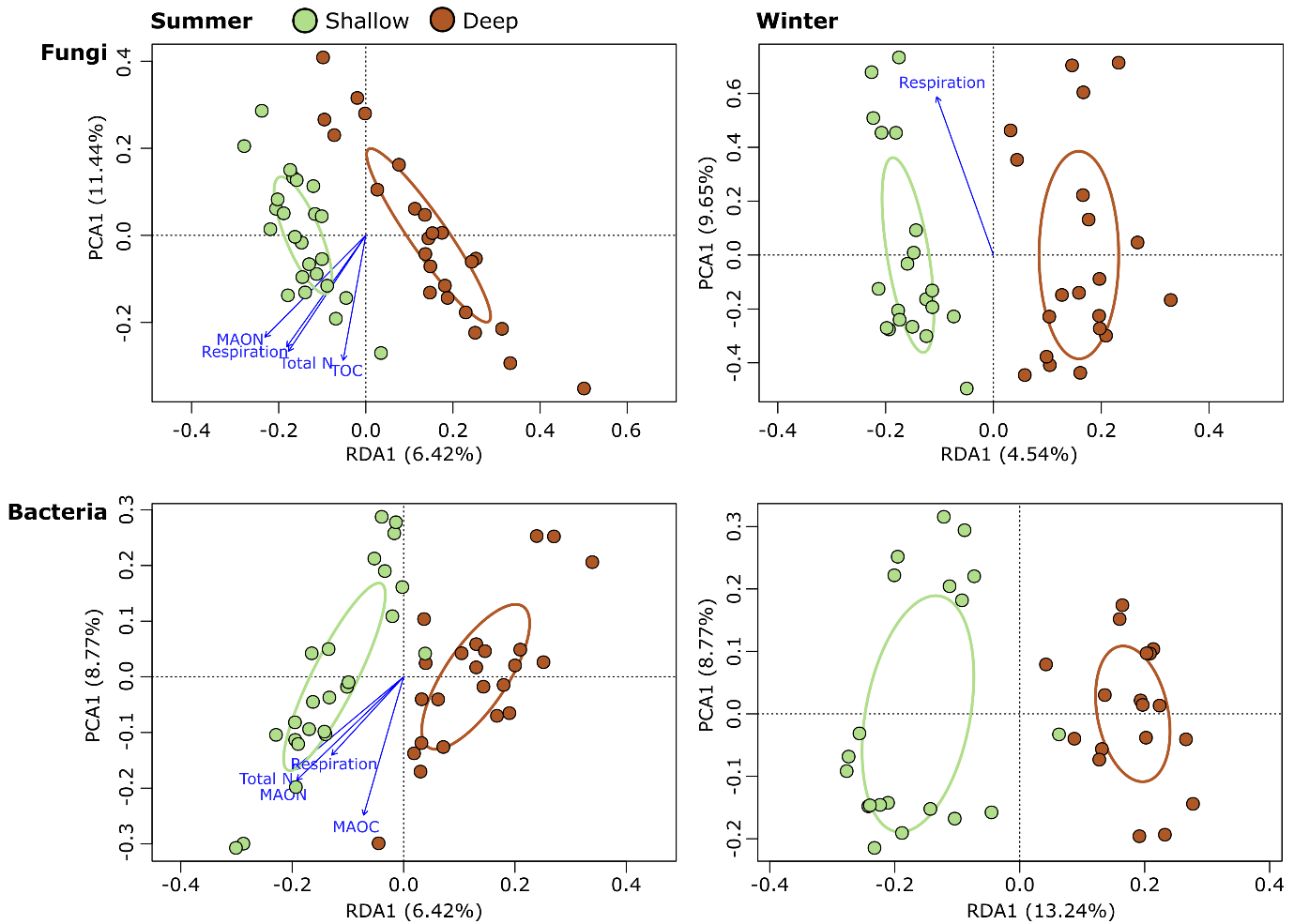


**Fig. S5** Redundancy analysis of the effect of sampling depth on the composition of bacterial and fungal OTUs in different summer and winter broadacre rotations. Ellipses represent standard deviations of centroids. Arrows represent significant correlations of community composition with abiotic variables, lengths of the arrows represent the strength of the correlation.


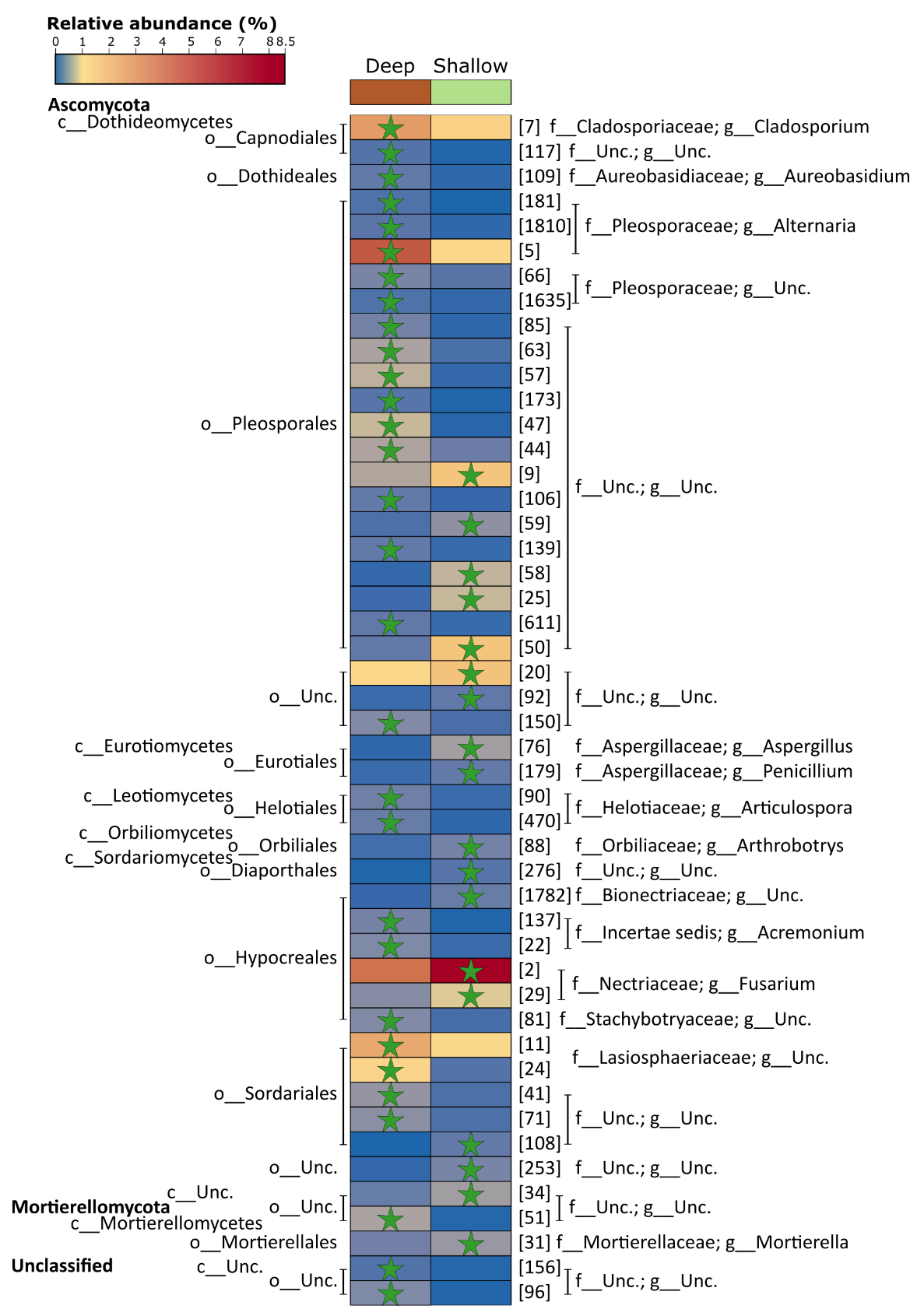


**Fig S6** A heatmap showing fungal indicator OTUs from different depth across all summer crop rotations. Tiles indicate mean relative abundances of OTUs within a rotation. Relative abundances between 0-1% are represented by a blue-yellow scale, those 1-8.5% are represented by a yellow-red scale. Green stars indicate which depth each OTU is an indicator of.


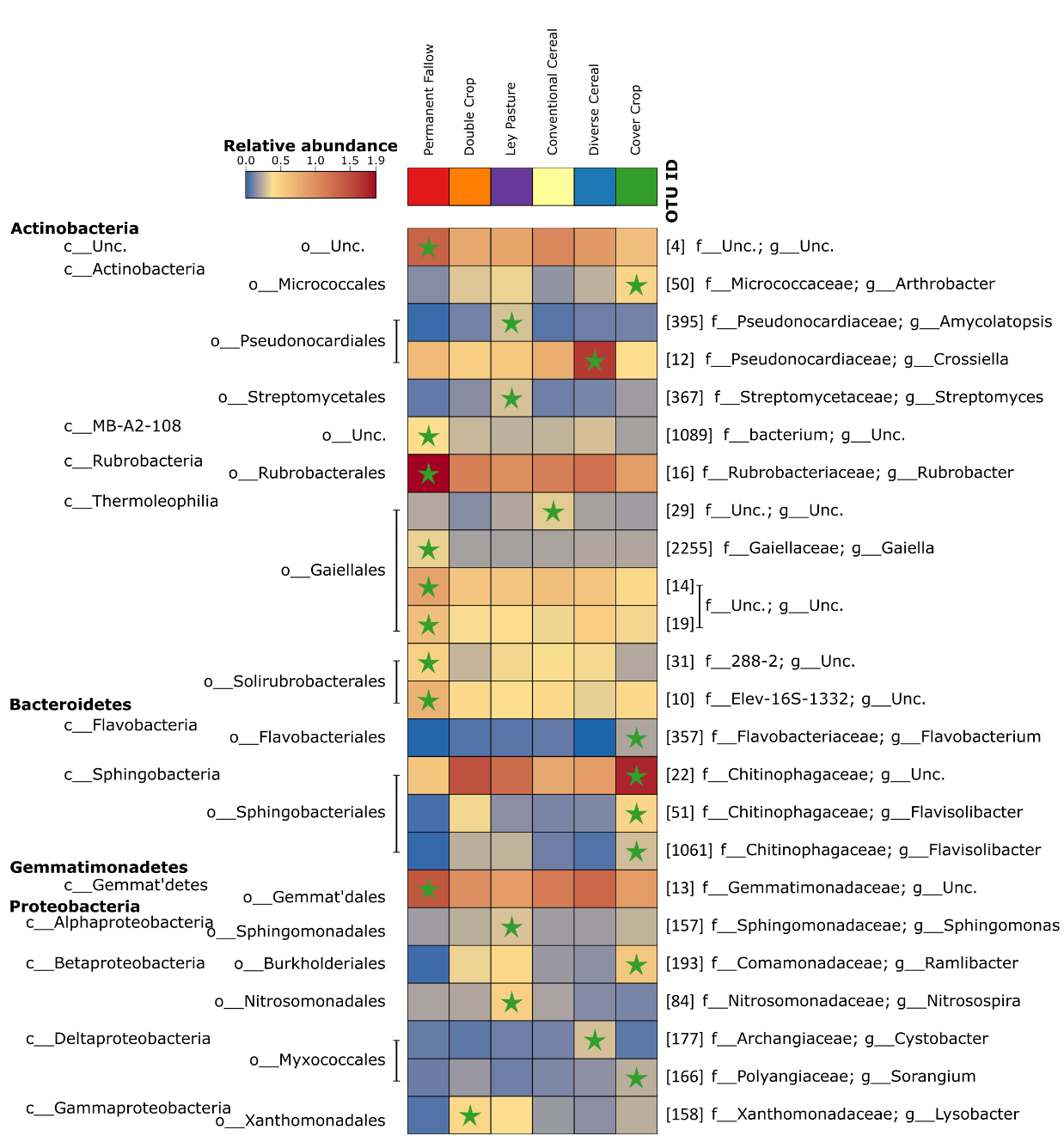


**Fig S7** A heatmap showing bacterial indicator OTUs from different summer crop rotations. Tiles indicate mean relative abundances of OTUs within a rotation. Relative abundances between 0-0.5% are represented by a blue-yellow scale, those 0.5-1.9% are represented by a yellow-red scale. Green stars indicate which rotation each OTU is an indicator of.


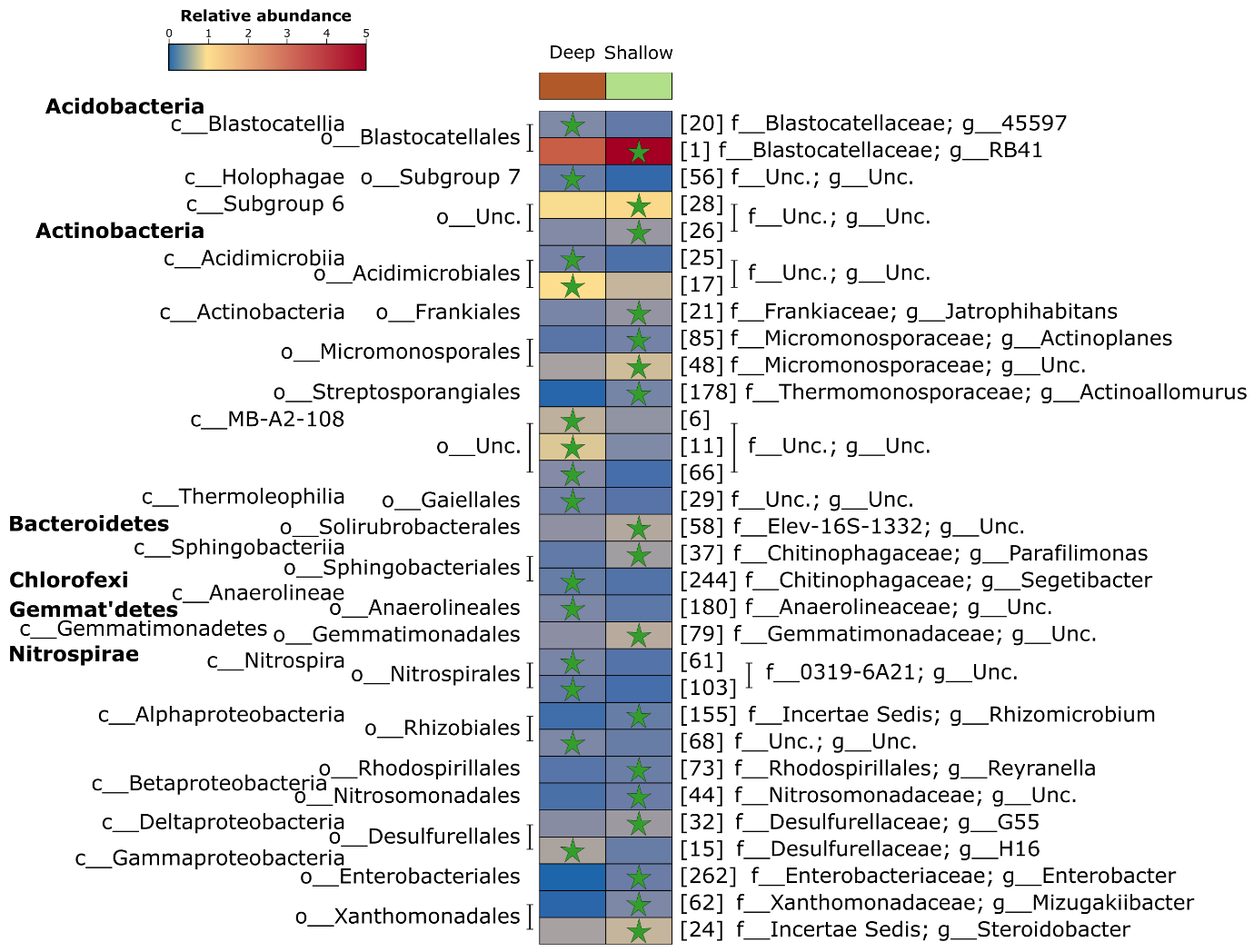


**Fig S8** A heatmap showing bacterial indicator OTUs from different depth across all summer crop rotations. Tiles indicate mean relative abundances of OTUs within a rotation. Relative abundances between 0-1% are represented by a blue-yellow scale, those 1-5% are represented by a yellow-red scale. Green stars indicate which depth each OTU is an indicator of.


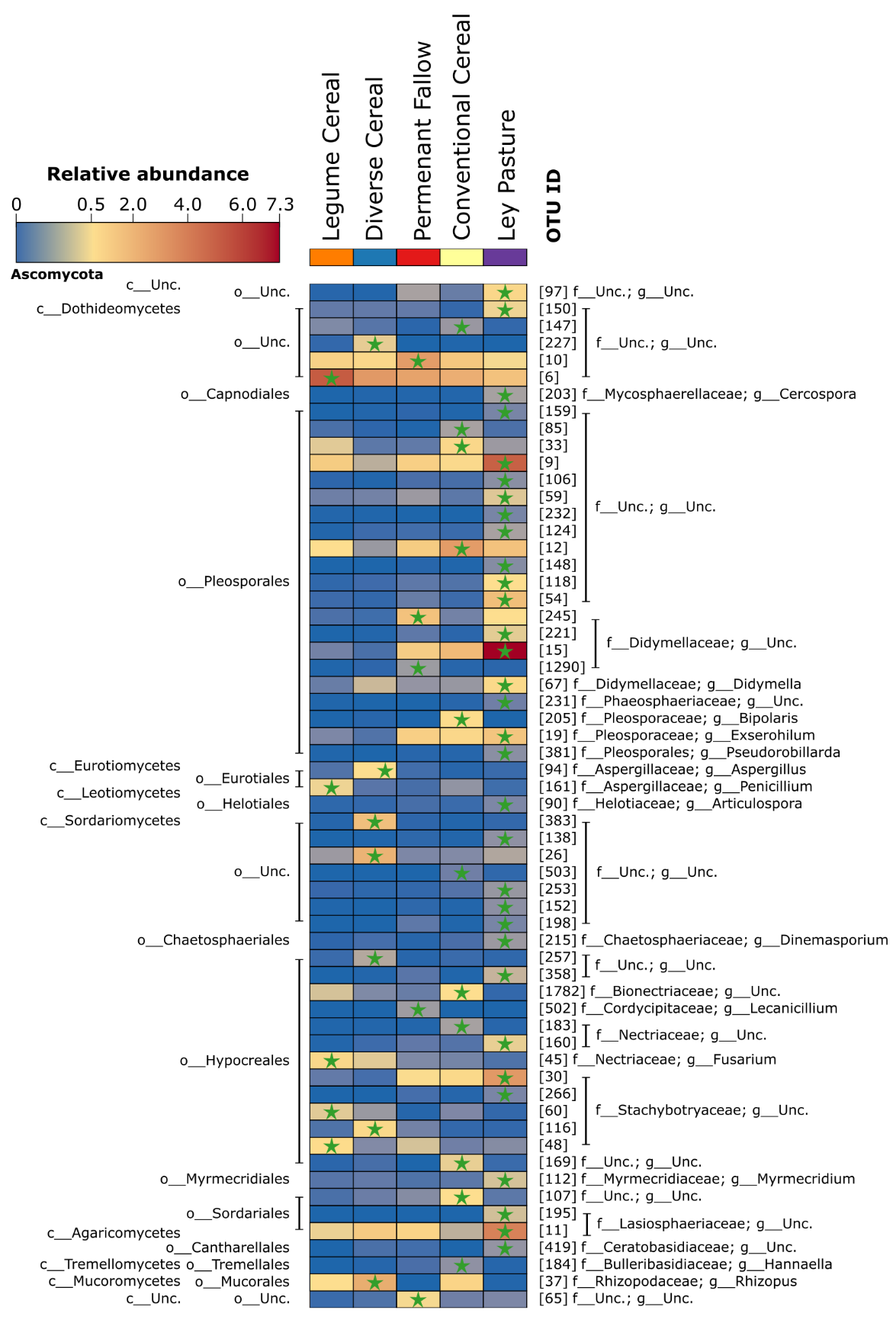


**Fig S9** A heatmap showing fungal indicator OTUs from different winter crop rotations. Tiles indicate mean relative abundances of OTUs within a rotation. Relative abundances between 0-0.5% are represented by a blue-yellow scale, those 0.5-7.3% are represented by a yellow-red scale. Green stars indicate which rotation each OTU is an indicator of.


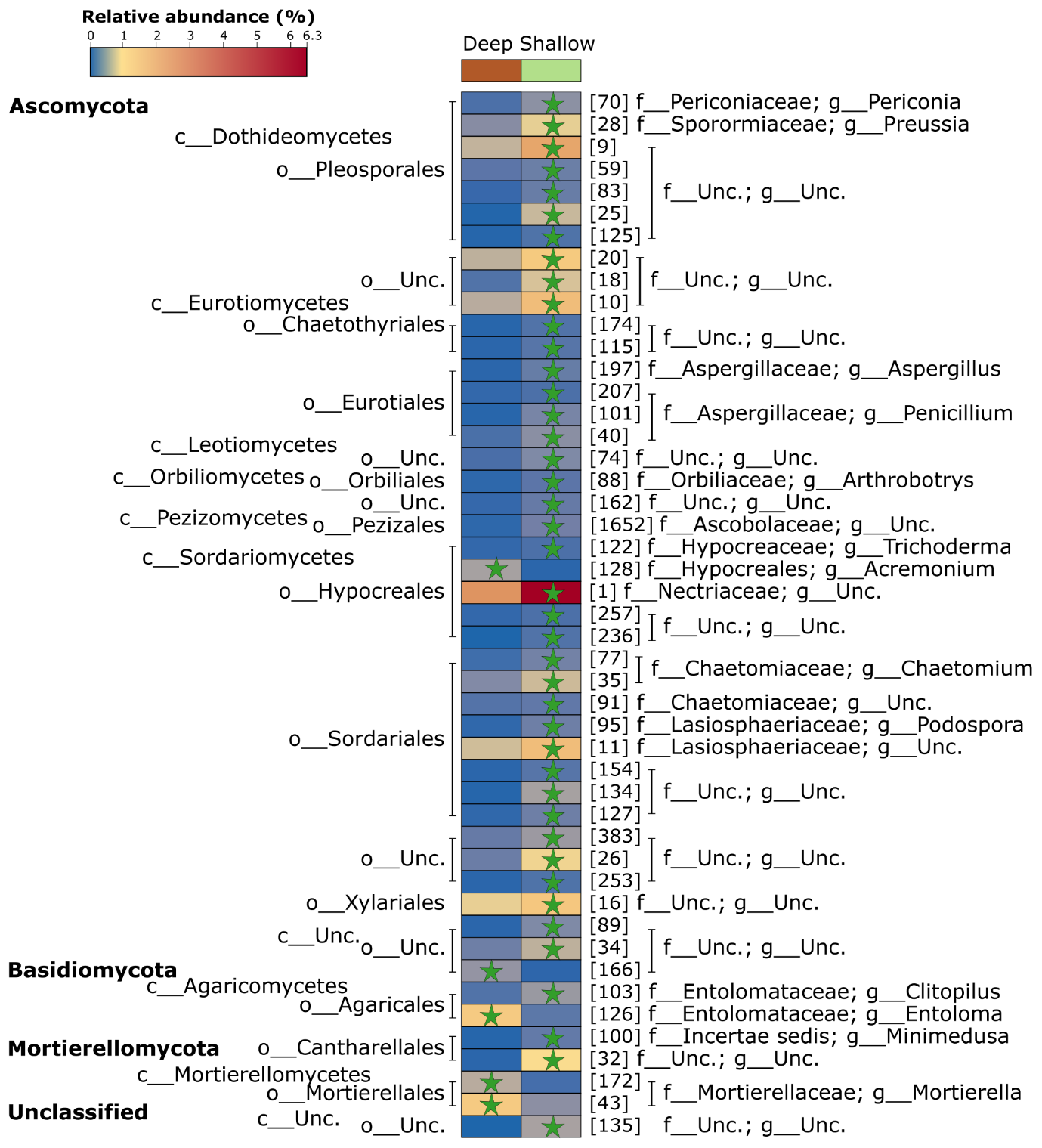


**Fig S10** A heatmap showing fungal indicator OTUs from different depth across all winter crop rotations. Tiles indicate mean relative abundances of OTUs within a rotation. Relative abundances between 0-1% are represented by a blue-yellow scale, those 1-6.3% are represented by a yellow-red scale. Green stars indicate which depth each OTU is an indicator of.


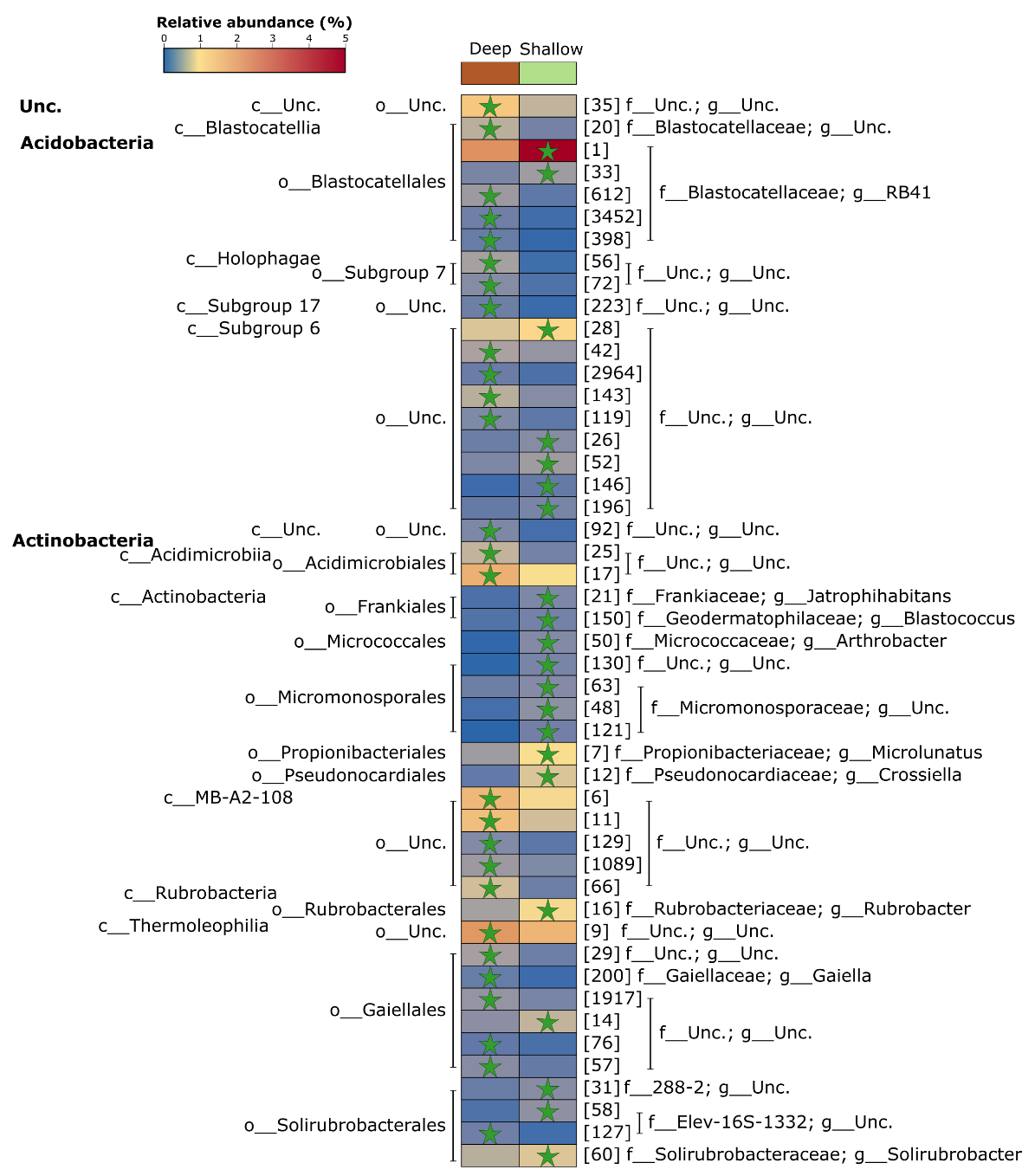


**Fig S11** A heatmap showing bacterial indicator OTUs from different depth across all summer crop rotations. Tiles indicate mean relative abundances of OTUs within a rotation. Relative abundances between 0-1% are represented by a blue-yellow scale, those 1-5% are represented

by a yellow-red scale. Green stars indicate which depth each OTU is an indicator of.


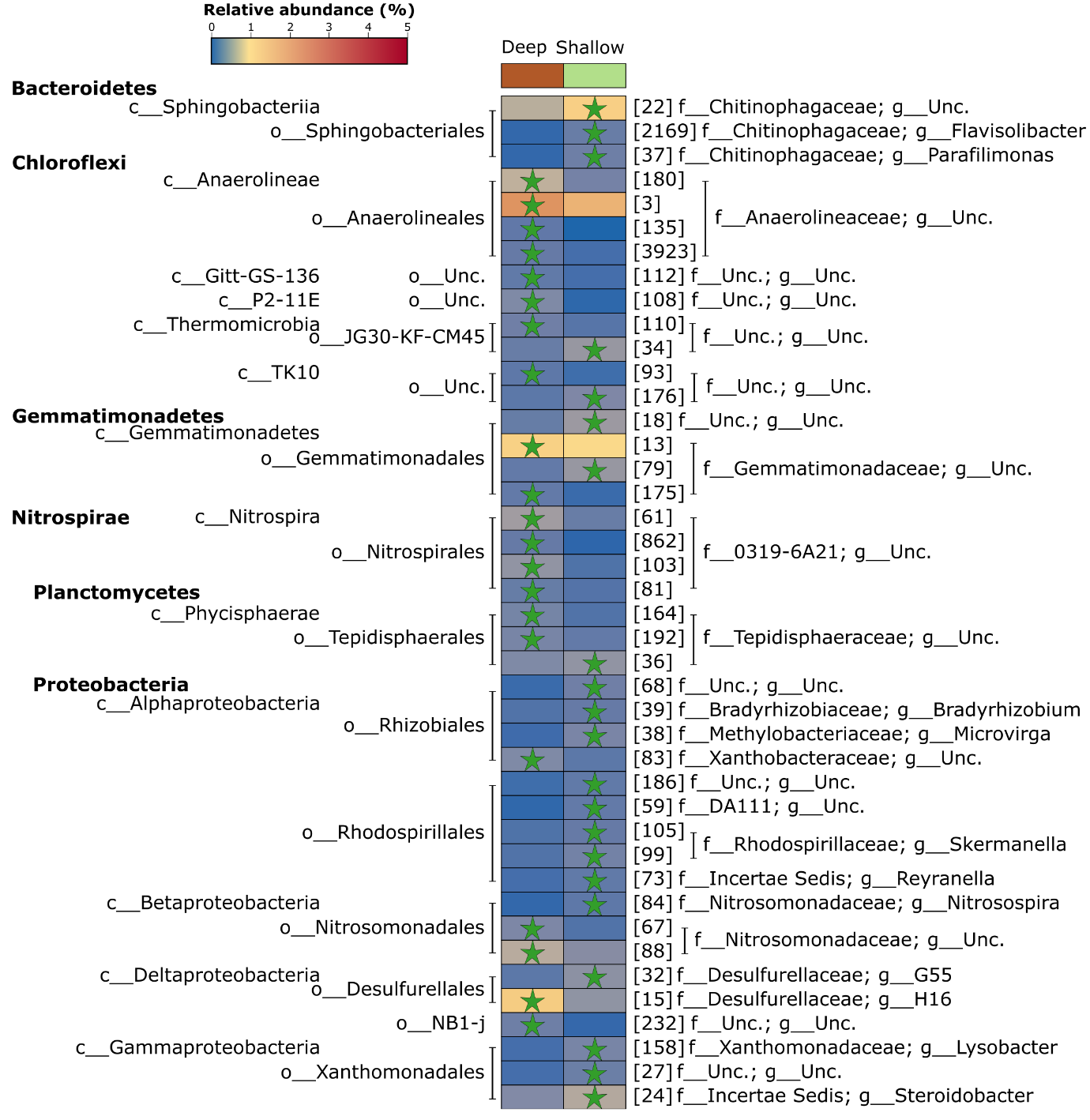


**Fig S12** A heatmap showing bacterial indicator OTUs from different depth across all summer crop rotations. Tiles indicate mean relative abundances of OTUs within a rotation. Relative abundances between 0-1% are represented by a blue-yellow scale, those 1-5% are represented

by a yellow-red scale. Green stars indicate which depth each OTU is an indicator of.
